# Noncontractible loop states from a partially flat band in a photonic borophene lattice

**DOI:** 10.1515/nanoph-2023-0222

**Published:** 2023-07-31

**Authors:** Philip Menz, Haissam Hanafi, Jörg Imbrock, Cornelia Denz

**Affiliations:** Institute of Applied Physics, University of Münster, Münster, Germany; Physikalisch-Technische Bundesanstalt (PTB), Bundesallee 100, 38116 Braunschweig, Germany

**Keywords:** borophene, compact localized states (CLS), flat band, noncontractible loop states (NLS), topological photonics, wave localization

## Abstract

Flat band systems are commonly associated with compact localized states (CLSs) that arise from the macroscopic degeneracy of eigenstates at the flat band energy. However, in the case of singular flat bands, conventional localized flat band states are incomplete, leading to the existence of noncontractible loop states (NLSs) with nontrivial real-space topology. In this study, we experimentally and analytically demonstrate the existence of NLSs in a 2D photonic borophene lattice without a CLS counterpart, owing to a band that is flat only along high-symmetry lines and dispersive along others. Our findings challenge the conventional notion that NLSs are necessarily linked to robust boundary modes due to a bulk-boundary correspondence. Protected by the band flatness that originates from band touching, NLSs play a significant role in investigating the fundamental physics of flat band systems.

## 1 Introduction

Flat band systems have attracted significant interest due to their quenched kinetic energy, which maximizes interactions and renders them an ideal playground to study strongly correlated phenomena such as the fractional quantum Hall effect [1] and superconductivity [2]. These systems are characterized by a flat band, which is a dispersionless energy band with vanishing wave group velocity at all momenta in the Brillouin zone. The presence of a flat band with its macroscopic degeneracy of eigenstates in the spectrum of a periodic lattice implies the existence of so-called compact localized states (CLSs). CLSs are wave functions that are strictly confined to a finite number of lattice sites and remain intact during evolution due to destructive interference. For a lattice of *N* unit cells, there should be *N* linearly independent CLSs to span the entire flat band. However, it has been shown that if the flat band has a singular band touching point with a dispersive band, the maximum number of linearly independent CLSs is always less than *N* [3, 4]. Missing states that complete the basis can be identified as the so-called noncontractible loop states (NLSs). These are states that extend indefinitely along a certain direction, while being localized along the others. They manifest nontrivial real space topology as they span closed loops that wind around the torus representing the lattice with periodic boundary conditions.

Until now, NLSs have always been associated with lattice systems that have flat bands over the entire Brillouin zone. In fact, their presence (absence) is the key signature of the singularity (nonsingularity) of a flat band [5–7]. Here, we break new ground and show that NLS are not only a feature of completely flat bands. In our photonic realization of a chiral borophene lattice, they arise from bands that are partially flat. Where with partially flat we mean that they are flat along high-symmetry lines in the in the Brillouin zone, while dispersive along other directions [8]. Consequently, this kind of flat band does not host any CLSs. Additionally, we reveal that the three NLSs of the chiral borophene lattice are linearly independent and wind around the torus in three topologically distinct directions. This is in stark contrast to previously reported cases where always only two linearly independent NLSs exist, like in the Lieb [9], kagome [10], or super-honeycomb lattice [11]. Also, what gives further relevance to our results is the fact that we propose and realize flat band states in the photonic analog of a realistic atomic 2D material, namely a borophene allotrope [12, 13].

The theoretical prediction of NLSs represents one aspect of the challenge, while the other aspect involves the direct experimental observation, which proves to be difficult since NLSs are stable only in an infinite lattice or under periodic boundary conditions. The latter could be realized in atomic borophene in the future, e.g., in the form of fullerene-like or carbon nanotube-like structures. However, the typical lattices considered in flat band experiments are usually finite with open boundaries. This is the case for artificial lattice realizations in the form of metamaterials [14], Bose–Einstein condensates [15], polariton condensates [16], acoustic lattices [17], and photonic waveguide arrays [18–22], as well as for 2D atomic materials such as the recently realized electronic Lieb and kagome lattices [23, 24]. A solution to this problem is to truncate the finite lattice with appropriately tailored boundaries that stabilize the NLSs in the form of line states. Recently, these line states have been realized in a photonic Lieb lattice [9], and a photonic super-honeycomb lattice [11]. Hence, we adopt the versatile technique of femtosecond direct laser writing which enables us to fabricate a finite photonic borophene lattice with tailored open boundaries to stabilize the NLSs. We employ this lattice to experimentally demonstrate the existence of three linearly independent NLSs originating from a partially flat band through their nondiffracting propagation in the form of structured light fields.

## 2 The chiral borophene photonic lattice and its partially flat band

**Figure 1:**
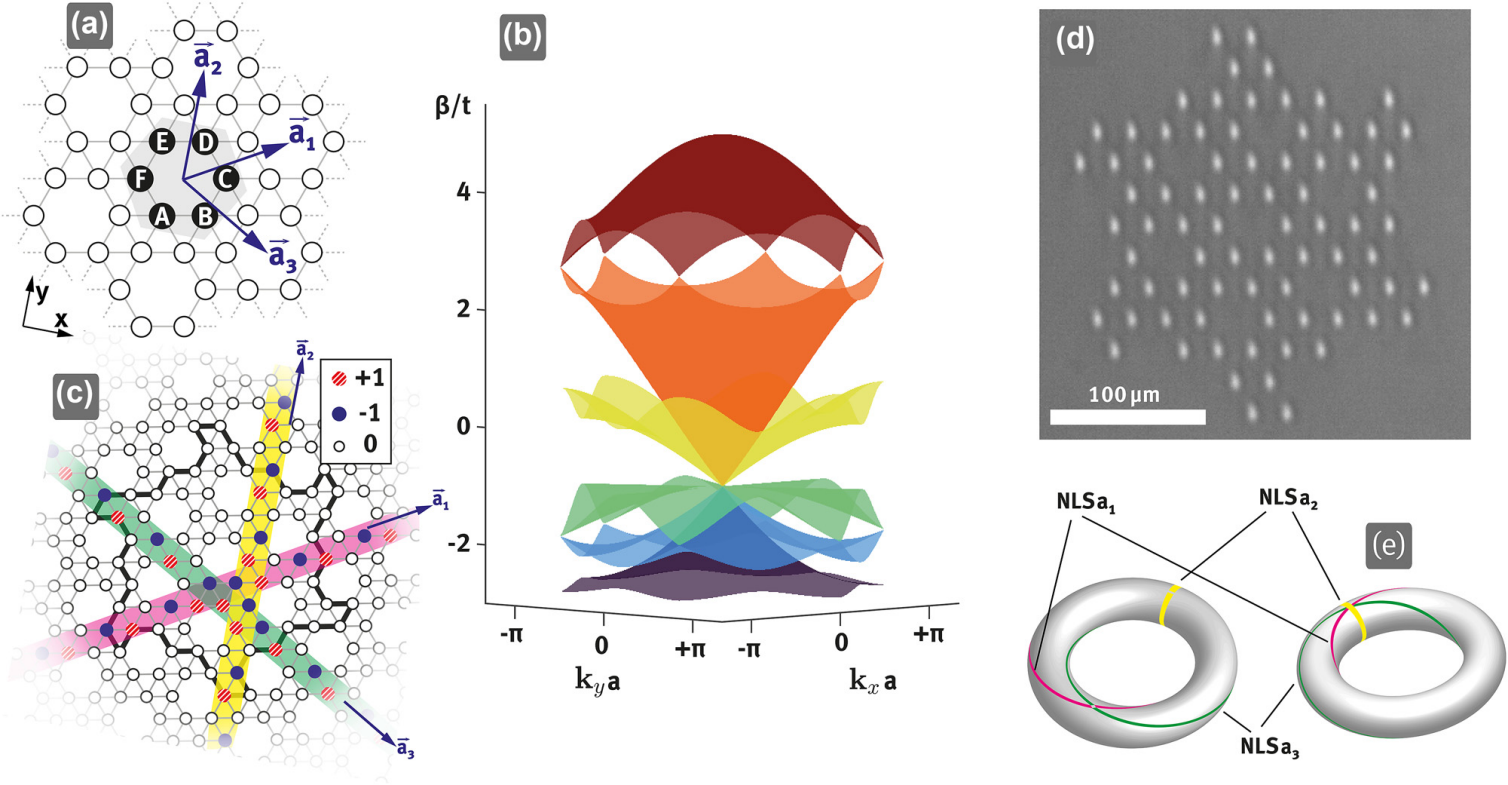
Geometry, band structure, and topological characteristics of the chiral borophene lattice. (a) Schematic of the chiral borophene lattice, with the unit cell highlighted in gray and the lattice sites labeled from A to F. (b) Tight-binding band structure in the first Brillouin zone. (c) The three linearly independent NLSs in an *infinite* lattice, the bold lines indicate the tailored boundaries that stabilize the NLSs in a finite lattice. Lattice sites with zero amplitude are indicated by empty circles, while filled blue and hatched red lattice sites indicate an equal amplitude but opposite phase. (d) Microscope image of the laser-written chiral borophene lattice in fused silica with tailored boundaries. (e) Different perspectives on NLSs winding a torus of periodic boundary conditions.

To demonstrate the three linearly independent NLSs arising from a partially flat band, we rely on the borophene lattice shown in Figure 1(a). In solid states physics, this lattice consisting of a planar sheet of boron atoms is known as the *χ*–*h*_0_ phase, snub, or chiral borophene [12, 13] and corresponds to an Archimedean tessellation. This so-called snub hexagonal tiling of the plane is composed of regular triangles and hexagons and exists in two chiral variants [25]. The hexagonal unit cell is composed of six lattice sites (A, B, C, D, E, and F) as marked in Figure 1(a). In a photonic waveguide realization of this lattice, the propagation of light therein is governed by a Schrödinger-type paraxial wave equation [26]. Under the tight-binding approximation, a discrete model describing the coupling between the lattice sites can be used. Considering nearest neighbor coupling only, we obtain a *k*-space Hamiltonian

(1) $${\hat{H}}_{k}=t\left(\begin{array}{cccccc} 0 & 1 & e^{-\text{i}a_{1}k} & e^{-\text{i}a_{2}k} & e^{-\text{i}a_{2}k} & 1\\ 1 & 0 & 1 & e^{-\text{i}a_{2}k} & e^{\text{i}a_{3}k} & e^{\text{i}a_{3}k}\\ e^{\text{i}a_{1}k} & 1 & 0 & 1 & e^{\text{i}a_{3}k} & e^{\text{i}a_{1}k}\\ e^{\text{i}a_{2}k} & e^{\text{i}a_{2}k} & 1 & 0 & 1 & e^{\text{i}a_{1}k}\\ e^{\text{i}a_{2}k} & e^{-\text{i}a_{3}k} & e^{-\text{i}a_{3}k} & 1 & 0 & 1\\ 1 & e^{-\text{i}a_{3}k} & e^{-\text{i}a_{1}k} & e^{-\text{i}a_{1}k} & 1 & 0 \end{array}\right),$$

where the lattice vectors are given by $a_{1}=a/2\left(\sqrt{3},1\right)$, **a**_**2**_ = *a*(0, 1), and **a**_**3**_ = **a**_**1**_ − **a**_**2**_, *a* is the lattice constant and *t* is the coupling strength. The eigenvalues of $\hat{H}$ give the spectrum *β*(**k**) shown in Figure 1(b). In the case of a photonic lattice, the band structure with its propagation constant *β*(**k**) represents a diffraction relation that describes the *spatial* evolution dynamics of photonic wave functions in the lattice. In an atomic borophene lattice, this would correspond to an energy spectrum describing the *temporal* evolution of the electronic wave function. Therefore, absence of dispersion in the electronic case corresponds to non-diffractive in the photonic one [27].

**Figure 2:**
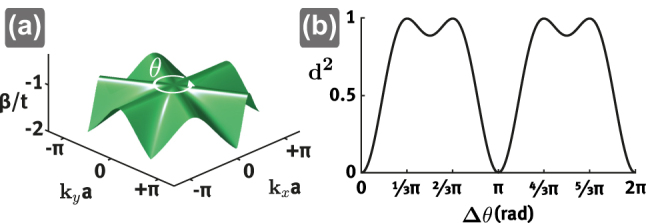
Hilbert–Schmidt quantum distance for two eigenstates of the partially flat band. (a) Arbitrarily close circle around the singular Γ-point. (b) Quantum distance as a function of the angle between two eigenstates on (a).

The band structure is composed of six bands of which five meet at the center of the Brillouin zone, i.e. the Γ-point, forming a pseudospin-2 conical intersection [28]. In this work, we focus on the middle band of this higher-order conical intersection, which is partially flat. As shown in Figure 2(a), the band is perfectly flat at *β* = −*t* along three high-symmetry lines in the Brillouin zone given by the reciprocal lattice vectors which meet at the singular Γ-point of fivefold degeneracy. There is an NLS belonging to each of these flat high-symmetry lines resulting from the band touching similarly to previously studied singular flat band lattices like the Lieb [29], or the kagome one [10]. However, as the band is not entirely flat, there exist no CLSs, and no combination of NLSs can lead to states localized along all directions in the lattice. This finding is in stark contrast to the previously described systems. Expressions for the NLSs can be obtained by reducing the 2D to a 1D Hamiltonian. As any 1D flat band has been shown to be nonsingular, it is always possible to find a state that is localized along this dimension and extends indefinitely along the perpendicular direction when going back to the full 2D Hamiltonian [4]. In this way, we can determine the three NLSs that extend unbounded along the lattice vectors **a**_1,2,3_, which are shown in Figure 1(c) (see Supplementary Material, Section I for details). These NLSs consist of lattice sites that have the same amplitude but are pairwise out-of-phase, satisfying the destructive interference condition that keeps them localized by preventing diffraction via coupling to other waveguides [30]. Remarkably, the three NLSs are linearly independent of each other, which means that it is not possible to obtain any one of them by combining the other two. This can be seen in Figure 1(c) from the fact that the NLSs reside at different lattice sites of the unit cell: A and D for the NLS extended along **a**_3_, B and E for the NLS along **a**_1_, and C and F for the NLS along **a**_2_. It is important to highlight that while the NLSs manifest as lines in the 2D lattice, the underlying vector space of the lattice is 6-dimensional. This higher-dimensional vector space allows for the possibility of more than two linearly independent NLSs. This is a new situation compared to NLSs that have been previously reported, which are always only independent in pairs [9–11] (see Supplementary Material, Section II for details). On a torus representing the lattice with periodic boundaries, as can be seen in Figure 1(e) and (f), the NLSs show a distinct real space topology in their winding around the torus. ${\text{NLS}}_{a_{2}}$ forms a loop along the poloidal direction (yellow), while ${\text{NLS}}_{a_{1}}$ and ${\text{NLS}}_{a_{3}}$ are in the form of Villarceau circles (magenta and green, respectively) (see Supplementary Material, Section III for details).

Further insight into the linear independence of the NLSs can be gained by considering the Hilbert–Schmidt quantum distance of the states of the partially flat band around the band touching singularity. This measure has recently been proposed as a bulk invariant to measure the strength of the singularity at the band crossing point [31]. The Hilbert–Schmidt quantum distance is defined as [32]

(2) $$d^{2}\left(\psi_{1},\psi_{2}\right)=1-{\left|\left\langle \psi_{1}|\psi_{2}\right\rangle \right|}^{2},$$

where *ψ*_1_ and *ψ*_2_ are normalized quantum states. For the quantum distance, we have 0 ≤ *d*^2^ ≤ 1, where a value of zero means that two quantum states are parallel, while a value of one means they are orthogonal. In our case, we are interested in the distance between eigenstates in momentum space. The quantum distance of two such eigenstates at **k**_1_ and **k**_2_ usually tends to zero for two close momenta $\left|k_{1}-k_{2}\right|\to 0$. However, around the singular band crossing point of a flat band with a dispersive one, two eigenstates develop a nonzero quantum distance even for really close momenta. In the following we calculate the quantum distance for a pair of eigenstates of our partially flat band on an arbitrarily small circle around the singular Γ-point with the polar angle *θ* at **k**_1_ = *ϵ*(cos*θ*_1_, sin*θ*_1_) and **k**_2_ = *ϵ*(cos*θ*_2_, sin*θ*_2_). For *ϵ* → 0, an eigenstate of the partially flat band of Eq. (1) reads

(3) $$|\psi \left(\theta \right)〉=\frac{\sqrt{2}}{6}\left(\begin{array}{c} 1+2\sin\left(2\theta -\pi /6\right)\\ -1+2\sin\left(2\theta +\pi /6\right)\\ 1+2\cos\left(2\theta \right)\\ -1-2\sin\left(2\theta -\pi /6\right)\\ 1-2\sin\left(2\theta +\pi /6\right)\\ -1-2\cos\left(2\theta \right) \end{array}\right).$$

From this, with Δ*θ* = *θ*_1_ − *θ*_2_, we get $\left\langle \psi_{1}|\psi_{2}\right\rangle =1/3+2/3\cos\left(2\Delta \theta \right)$, and therefore the quantum distance shown in Figure 2, which is given by

(4) $$d^{2}\left(\Delta \theta \right)=\frac{1}{9}\left[8-4\cos\left(2\Delta \theta \right)-4\cos^{2}\left(2\Delta \theta \right)\right].$$

We can see that any two eigenstates are orthogonal to each other at Δ*θ* = *π*/3 + *nπ* and Δ*θ* = 2/3*π* + *nπ* with n∈Z. There the quantum distance reaches its maximum value of one. The NLSs are constructed by eigenstates along the high-symmetry lines which are located at Δ*θ* = *π*/3 and Δ*θ* = 2/3*π* from each other. Since the eigenstates on each line are orthogonal to those on the other two, the NLSs are linearly independent. The presence of three orthogonal eigenstates around the degeneracy is a novel situation compared to previously reported singular flat bands where only two of them exist [5].

It is important to emphasize that the task of constructing a 2D lattice with multiple NLSs is not straightforward. Trying to combine multiple 1D lattices with CLSs and just extending those CLSs along the directions perpendicular to the localization will often destroy the flatness of the band. If the flatness is preserved, one normally obtains a completely flat band and not a partial one. Of course, in terms of analytical solutions a lot is conceivable, and by properly designing the Hamiltonian (complex-, negative-, non-local-coupling etc.) it should be possible to freely tailor the characteristics of the flat bands. However, the NLSs without CLS counterparts shown here rely solely on reasonable geometric nearest-neighbor coupling in a relatively simple lattice structure that has real-world relevance in the form of a borophene allotrope.

## 3 Waveguide fabrication in fused silica

To experimentally demonstrate the existence of the three NLSs, we fabricate a sample composed of 78 single-mode waveguides arranged in chiral borophene lattice geometry. The waveguides extend for 2 cm in the propagation direction *z* and are induced by femtosecond direct laser writing in fused silica. The fabrication process is carried out with the same parameters reported in Ref. [6]. Waveguides induced by direct laser writing exhibit a slight ellipticity leading to anisotropic coupling in vertical and horizontal direction [33]. To reduce this ellipticity, we employ a slit beam shaping technique and simultaneously correct the aberrative wave front using a spatial light modulator (SLM) [34, 35]. The coupling strength in our photonic lattice system is determined by the overlap of the evanescent fields of the modes in the waveguides. This can be tailored by adjusting several parameters, including the separation between the waveguides, the refractive index modulation, and the wavelength of the exciting laser beam. To obtain isotropic nearest neighbor coupling, we slightly increase the vertical separation distance of the waveguides [36]. Since the coupling follows an exponential decaying law on the distance, with a vertical stretch factor of 1.05 we are able to obtain nearly equal coupling strength of *t* ≈ 0.5 cm^−1^ for horizontal and diagonal coupling between waveguides. However, it is important to note that the partially flat band observed in our lattice model arises from local symmetry, rather than from finely tuned couplings. Because of this, our experiments are not critically dependent on the specific wavelength of the laser beam. In principle, the experiments could be carried out at different wavelengths, provided that the waveguides remain single-mode and the coupling strength is sufficiently high to enable observable interactions. This flexibility further underscores the robustness and versatility of our photonic borophene lattice system. A microscope image of the front facet of the fabricated sample is shown in Figure 1(d). To ensure sufficient coupling during propagation in the sample while minimizing unwanted next-nearest neighbor coupling, we choose a horizontal waveguide separation distance of Λ = 22 μm which corresponds to a lattice constant $a=\sqrt{7}\Lambda$. To stabilize the NLSs in a finite open boundary lattice, where by stabilizing we mean making them exact eigenstates, we choose the appropriately tailored termination shown in Figure 1(d). Due to the periodicity of the lattice a demonstration of line states in a finite lattice is evidence for the existence of NLSs in the infinite lattice. An indirect demonstration of the NLSs in the form of so-called robust boundary modes (RBMs) is not possible in our case since no CLSs exist and RBMs and CLSs are related by a bulk-boundary correspondence [10].

## 4 Experimental observation of NLS

**Figure 3:**
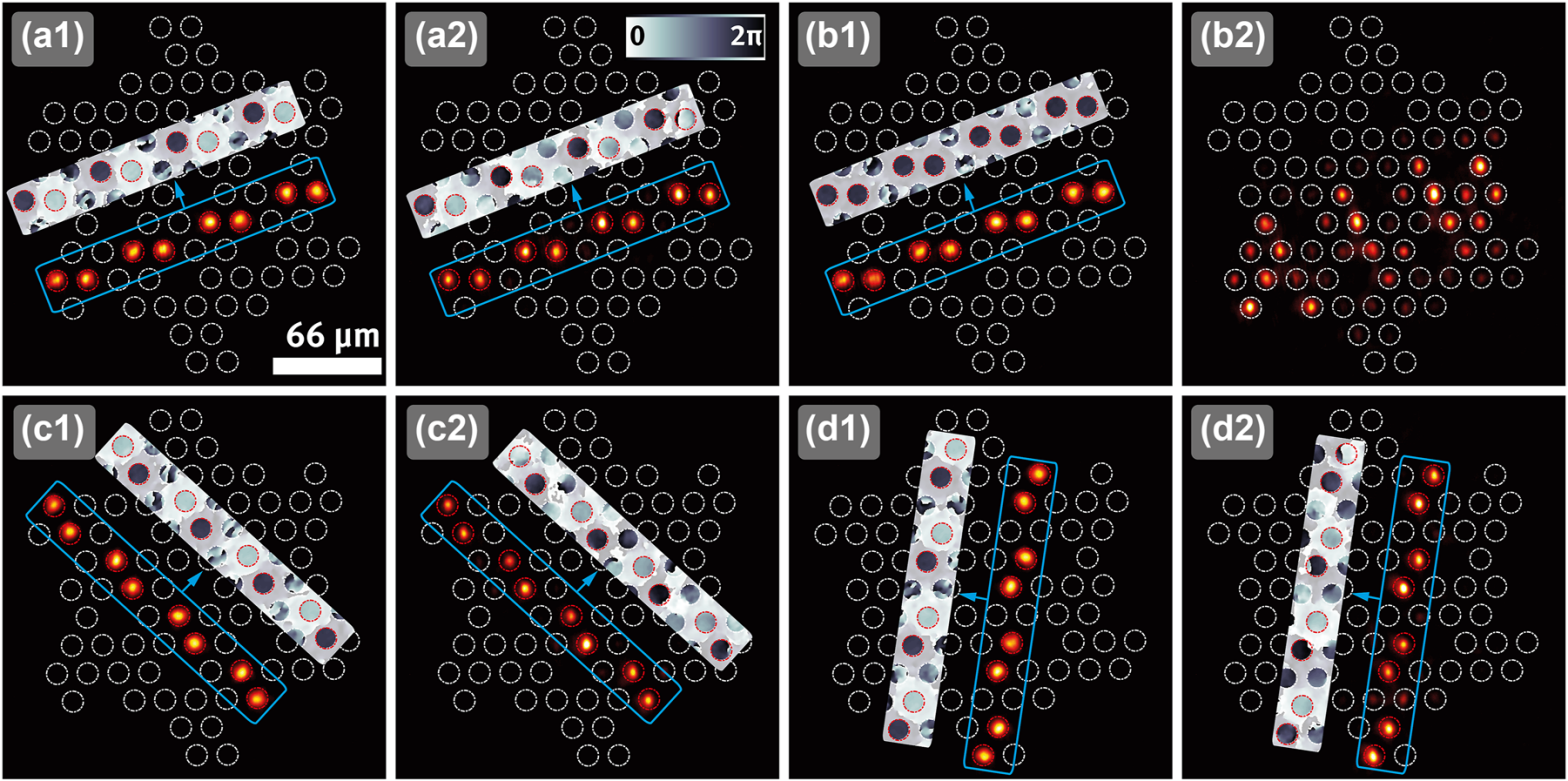
Experimental demonstration of diffractionless propagation of the NLSs in the chiral borophene photonic lattice. (a1) Intensity of the ${\text{NLS}}_{a_{1}}$ input light field with the phase shown in the inset. (a2) Same as (a1) but after propagation through the photonic lattice. (b1)–(b2) Same as (a1)–(a2), but for a diffracting state which has equal phase at every lattice site. (c1)–(c2) Same as (a1)–(a2), but for ${\text{NLS}}_{a_{3}}$. (d1)–(d2) Same as (a1)–(a2), but for ${\text{NLS}}_{a_{2}}$.

To observe the nondiffracting propagation of the NLSs in the photonic lattice, we shape a laser beam (*λ* = 532 nm) in the form of the NLSs by generating Gaussian spots of alternating phase with an SLM. The spots are arranged to match the positions of the waveguides corresponding to the NLSs. The first example light field corresponding to ${\text{NLS}}_{a_{1}}$ is shown in Figure 3(a1). As can be seen in the output recorded after propagation through the lattice in Figure 3(a2), the NLS stays completely localized during propagation in the photonic lattice as the destructive interference prevents it from diffracting. The out-of-phase relation is perfectly preserved as shown in the insets obtained by a digital holographic interference measurement [37]. For comparison, the results for an input of in-phase spots are shown in Figure 3(b1) and (b2). In this case, because the input light field is not a flat band one but is composed of modes from dispersive bands, there is considerable diffraction during propagation by coupling to previously unexcited waveguides. We then proceed to demonstrate the two remaining NLSs experimentally. The input and output light fields of ${\text{NLS}}_{a_{3}}$ are shown in Figure 3(c1) and (c2), respectively. Again, after propagation through the lattice, the intensity stays completely localized in the initially excited waveguides, as the out-of-phase relation is perfectly preserved. The results for ${\text{NLS}}_{a_{2}}$ are shown in Figure 3(d1) and (d2). They thoroughly confirm the expectation of diffractionless propagation inherent to a flat band state. In our experimental realization we are able to confirm the nondiffracting properties of the NLSs over the whole propagation distance of 2 cm of our fabricated lattice. In a numerical simulation (Supplementary Material, Section V) we show that the NLSs stably propagate without diffraction even for much longer propagation distances of up to 8 cm. With these observations we are able to confirm the existence of NLSs originating from a partially flat band without CLSs. An additional remarkable novelty of the NLSs of the chiral borophene lattice is that three of them are linearly independent of each other. This can be seen from the fact that they reside in different waveguides of the unit cell of the photonic lattice. The three observed linearly independent NLSs extend along different directions in the lattice. In a periodic boundary picture, one could see that they exhibit a distinct real space topological winding around the torus. Although we do not access the topological properties directly in our experiments, together with the analytical treatment of the NLSs (Supplementary Material, Section I and Section III), we provide strong evidence for them [10].

## 5 Conclusions

In conclusion, the results presented here advance the current understanding of wave localization in flat band systems by demonstrating that NLSs are not exclusively a feature of completely flat bands. The NLSs we observed originate from a band that is only partially flat along three high-symmetry lines in the Brillouin zone that meet at a fivefold degenerate conical intersection. Due to the non-flatness of the whole band, there exist no CLSs, and consequently, no robust boundary modes can be found. Our work thus opens up new avenues for studying unconventional wave localization in lattices that are not usually considered flat band systems but have bands that remain flat along high-symmetry lines [8]. A crucial and new feature of our NLSs is the linear independence of three of them, meaning that they exhibit a distinct nontrivial real space topological winding. The realization of NLSs in a photonic version of a borophene allotrope presents exciting new possibilities for flat band physics in atomic 2D materials beyond artificial lattice platforms. Moreover, having demonstrated these states in a photonic system could be of relevance for more light-based flat band implementations in the form of lasers [38] and topological insulators [39–41]. Our results may prove relevant to similar related phenomena in an electronic setting. Thus being of relevance for the field of electronic flat band systems and highly correlated states.

## Supplementary Material

Supplementary Material Details
