# Supplementary material for: Noncontractible loop states from a partially flat band in a photonic borophene lattice
Source: Nanophotonics. 2023 Jul 31;12(17):3409–15. doi: 10.1515/nanoph-2023-0222 (PMC11501999; doi:10.1515/nanoph-2023-0222)
Supplement: Supplementary file 1 — Supplementary Material Details [file j_nanoph-2023-0222_suppl_001.pdf]

Supplementary Material: Noncontractible loop states from a partially flat band in a photonic borophene lattice

Philip Menz,[*,†] Haissam Hanafi,[*,†] Jörg Imbrock,[†] and Cornelia Denz[†,‡]

†*Institute of Applied Physics, University of Münster, Germany*

‡*Physikalisch-Technische Bundesanstalt (PTB), Bundesallee 100, 38116 Braunschweig, Germany*

E-mail: philip.menz@uni-muenster.de; haissam.hanafi@uni-muenster.de

I. Analytical derivation of the NLSs

Here we illustrate the analytical derivation of the NLSs belonging to the singular and partially flat band of the chiral borophene lattice. We start by reducing the 2D Hamiltonian of Eq. (1) to an effective 1D one along the directions of the reciprocal lattice vectors $\mathbf{b_1} = \frac{4\pi}{a\sqrt{3}}(1,0)$, $\mathbf{b_2} = \frac{4\pi}{a\sqrt{3}}(-1/2, \sqrt{3}/2)$ and $\mathbf{b_3} = \mathbf{b_1} + \mathbf{b_2}$. Along these directions the band is a 1D flat band and as such is guaranteed to be nonsingular.[1] It is therefore always possible to find a combination of Bloch wave functions giving a CLS. Returning to the 2D lattice, we keep the found state that is compact localized along the first direction and extend it periodically along the perpendicular direction, leading to the formation of an NLS.

We start by considering the $\mathbf{b_1}$-direction. We fix $\mathbf{a_2} = 0$ and get $\mathbf{a_1} = \mathbf{a_3}$. The Hamilto-

nian becomes

$$\hat{H}(\mathbf{k}) = t \begin{pmatrix} 0 & 1 & e^{-\mathrm{i}\mathbf{a}_1\mathbf{k}} & 1 & 1 & 1 \\ 1 & 0 & 1 & 1 & e^{\mathrm{i}\mathbf{a}_1\mathbf{k}} & e^{\mathrm{i}\mathbf{a}_1\mathbf{k}} \\ e^{\mathrm{i}\mathbf{a}_1\mathbf{k}} & 1 & 0 & 1 & e^{\mathrm{i}\mathbf{a}_1\mathbf{k}} & e^{\mathrm{i}\mathbf{a}_1\mathbf{k}} \\ 1 & 1 & 1 & 0 & 1 & e^{\mathrm{i}\mathbf{a}_1\mathbf{k}} \\ 1 & e^{-\mathrm{i}\mathbf{a}_1\mathbf{k}} & e^{-\mathrm{i}\mathbf{a}_1\mathbf{k}} & 1 & 0 & 1 \\ 1 & e^{-\mathrm{i}\mathbf{a}_1\mathbf{k}} & e^{-\mathrm{i}\mathbf{a}_1\mathbf{k}} & e^{-\mathrm{i}\mathbf{a}_1\mathbf{k}} & 1 & 0 \end{pmatrix}. \tag{S1}$$

The eigenvector for the third band, which is flat at $\beta = -t$ then reads

$$\mathbf{u}_{\mathbf{k}} = \frac{1}{\sqrt{2}} \begin{pmatrix} 0 \\ 0 \\ e^{\mathrm{i}\mathbf{a}_1\mathbf{k}} \\ 0 \\ 0 \\ -1 \end{pmatrix}. \tag{S2}$$

To obtain an expression for the CLS, we can perform an inverse Fourier transform of the eigenvector.[1] Around the unit cell located at $\mathbf{R} = m_1\mathbf{a_1} + m_2\mathbf{a_2} = 0$ we get

$$\mathbf{U}_{0,\mathbf{R}} = \frac{1}{\sqrt{2}} \begin{pmatrix} 0 \\ 0 \\ \delta_{\mathbf{R},-\mathbf{a}_1} \\ 0 \\ 0 \\ -\delta_{\mathbf{R},0} \end{pmatrix}, \tag{S3}$$

where the qth entry of the vector represents the amplitude at the qth lattice site (A to F). Returning to the 2D lattice, we have to repeat this expression unchanged along $\mathbf{a}_2$ to obtain an NLS extended along this direction. By repeating the procedure, but first fixing $\mathbf{a}_3 = 0$

and then $\mathbf{a}_1 = 0$, we obtain the following three (unnormalized) NLSs expressions

$$\mathrm{NLS}_{\mathbf{a}_2} = \sum_{n=0}^{N_{\mathbf{a}_2}-1} \begin{pmatrix} 0 \\ 0 \\ \delta_{\mathbf{R}+n\mathbf{a}_2,-\mathbf{a}_1+n\mathbf{a}_2} \\ 0 \\ 0 \\ -\delta_{\mathbf{R}+n\mathbf{a}_2,0+n\mathbf{a}_2} \end{pmatrix}, \tag{S4}$$

$$\mathrm{NLS}_{\mathbf{a}_3} = \sum_{n=0}^{N_{\mathbf{a}_3}-1} \begin{pmatrix} \delta_{\mathbf{R}+n\mathbf{a}_3,-\mathbf{a}_2+n\mathbf{a}_3} \\ 0 \\ 0 \\ -\delta_{\mathbf{R}+n\mathbf{a}_3,0+n\mathbf{a}_3} \\ 0 \\ 0 \end{pmatrix}, \tag{S5}$$

$$\mathrm{NLS}_{\mathbf{a}_1} = \sum_{n=0}^{N_{\mathbf{a}_1}-1} \begin{pmatrix} 0 \\ -\delta_{\mathbf{R}+n\mathbf{a}_1,0+n\mathbf{a}_1} \\ 0 \\ 0 \\ \delta_{\mathbf{R}+n\mathbf{a}_1,\mathbf{a}_3+n\mathbf{a}_1} \\ 0 \end{pmatrix}, \tag{S6}$$

where $N_{\mathbf{a}_{1,2,3}}$ stands for the number of unit cells in the respective directions. We can see that the three NLSs, having their amplitudes in different lattice sites of the unit cell, are orthogonal to each other and therefore also linearly independent.

II. Linear independence of the NLSs in the Lieb and kagome lattices

As shown in the previous section, a distinctive feature of the NLS of the chiral borophene lattice is the linear independence of three of them. For previously studied lattices with NLSs, only two linearly independent ones were found to exist. This is related to the Hilbert-Schmidt quantum distance which reaches a maximum value of one for orthogonal eigenstates on a singular flat band around the band touching point.

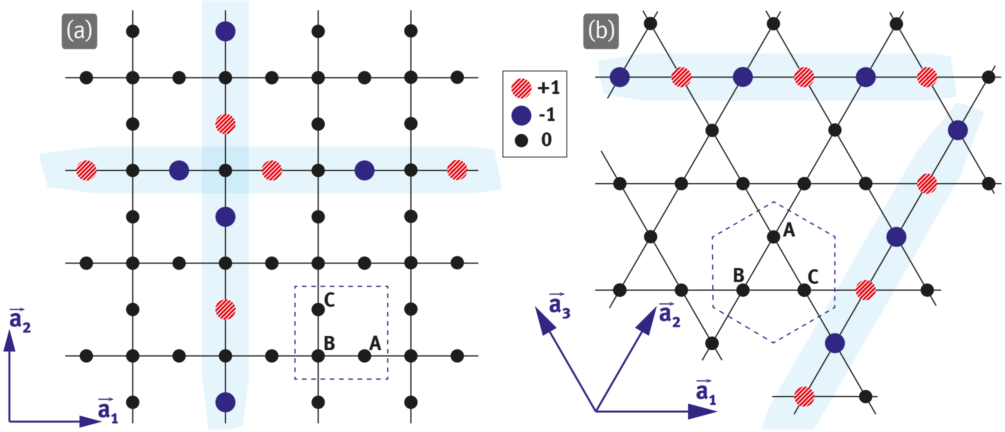

FIG. S1: NLSs of the (a) Lieb and (b) kagome lattice. Unit cell with lattice sites A to C highlighted by a dashed (a) square and (b) hexagon.

We want to exemplify this for the Lieb and the kagome lattice. The two NLSs of the Lieb lattice have their amplitude on different lattice sites of the unit cell (A and C sites respectively in FIG. S1 (a)). They are therefore not only linearly independent but also orthogonal like those of the chiral borophene lattice. For the kagome lattice, the two NLSs shown in FIG. S1 (b) are linearly independent but not orthogonal. The NLS along $\mathbf{a_1}$ has amplitude in the lattice sites B and C, while for the NLS along $\mathbf{a_2}$ the lattice sites are A and B. A third NLS along the $\mathbf{a_3}$-direction with amplitude in the lattice sites A and C is a linear combination of the other two. An eigenstate on the flat band of the Lieb lattice around and

close to ($\epsilon \to 0$) the degenerate M-point at $(\pi/a, \pi/a)$ at a polar angle θ is given by

$$\mathbf{v}_\theta = \begin{pmatrix} \sin(\theta) \\ 0 \\ -\cos(\theta) \end{pmatrix}. \tag{S7}$$

An eigenstate on the flat band of the kagome lattice around and close to ($\epsilon \to 0$) the degenerate Γ-point at $(0, 0)$ at a polar angle θ is given by

$$\mathbf{w}_\theta = \frac{2}{\sqrt{6}} \begin{pmatrix} -\cos(\theta) \\ \sin\left(\frac{\pi}{6} - \theta\right) \\ \sin\left(\frac{\pi}{6} + \theta\right) \end{pmatrix}. \tag{S8}$$

For the Hilbert-Schmidt quantum distance between two eigenstates at arbitrary angles θ and θ' with $\Delta\theta = \theta - \theta'$ one obtains the same result for the flat bands of both the Lieb and the kagome lattice

$$d^2_{\theta,\theta'} = 1 - |\langle \mathbf{v}_\theta \,|\, \mathbf{v}_{\theta'} \rangle|^2 = 1 - |\langle \mathbf{w}_\theta \,|\, \mathbf{w}_{\theta'} \rangle|^2 = \sin^2 \Delta\theta. \tag{S9}$$

Contrary to the case of the borophene lattice in the main manuscript, the quantum distance has a maximum of one only once between $\Delta\theta = 0$ and π. This means that around the singular band touching point, the eigenstates are orthogonal in pairs leading to the existence of two linearly independent NLSs.

III. Nontrivial real space topology of the NLSs

The nontrivial real space topology of the NLSs can be illustrated by considering the lattice with periodic boundaries. We can cut out a rectangular patch of the lattice that periodically repeats itself, like the one shown in FIG. S2 (a). If we join the opposite sides, first bottom and top and then left and right, we obtain a torus around which the NLSs have a nontrivial

winding. If we choose the orientation of the lattice and rectangular section shown in FIG. S2 (a), $NLS_{\mathbf{a_2}}$ winds around the torus in poloidal direction, while $NLS_{\mathbf{a_1}}$ and $NLS_{\mathbf{a_3}}$ are in form of Villarceau circles. This configuration is not unique, joining first the left and right side and then the bottom and top, one would obtain $NLS_{\mathbf{a_2}}$ in toroidal direction. Moreover, we could have chosen a different rectangular section, leading to a different winding direction of the NLSs. However, the three NLSs would always wind the torus in different ways, meaning that they possess distinct real space topology.

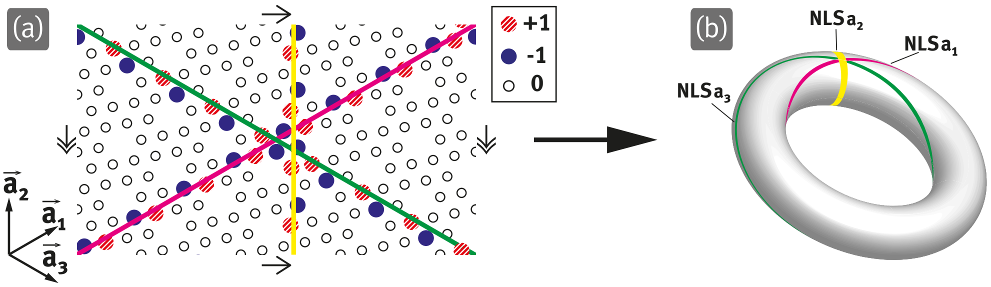

FIG. S2: Nontrivial real space topology of the NLSs. (a) Rectangular periodic patch of the lattice with NLSs. The arrows indicate which sides to join and in which order (first simple then double arrows) to obtain periodic boundary conditions. (b) Torus of periodic boundaries resulting from joining the sides of (a) with NLSs winding the torus in distinct ways.

IV. Experimental propagation of the diffracting counterparts to $NLS_{\mathbf{a_3}}$ and $NLS_{\mathbf{a_2}}$

For the sake of completeness we provide in the following additional experimental evidence that also $NLS_{\mathbf{a_3}}$ and $NLS_{\mathbf{a_2}}$ are nondiffracting flat band states. Exciting the lattice with light fields having the same Intensity profiles as the NLSs but with equal phase at every lattice site, we observe strong diffraction into the bulk of the lattice as depicted in FIG. S3. This observation is analogous to the results presented for $NLS_{\mathbf{a_1}}$ in Fig. 3(b1)-(b2) of the main manuscript. By qualitatively comparing the diffracted output patterns, and noticing their similarity, we are able to confirm the spatial homogeneity in coupling for the waveguides of

our lattice.

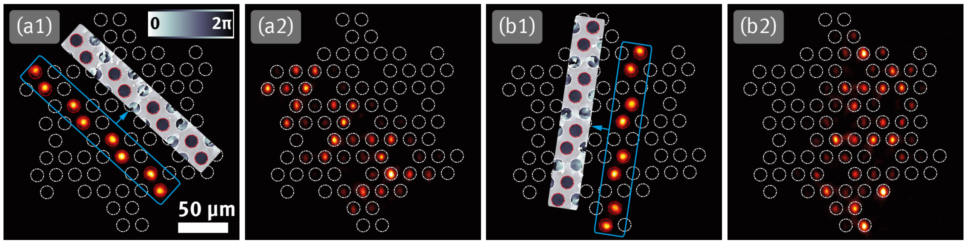

FIG. S3: Experimental demonstration of diffracting counterparts to $\mathrm{NLS}_{\mathbf{a_3}}$ and $\mathrm{NLS}_{\mathbf{a_2}}$. (a1) Intensity of the input light field having the same intensity as $\mathrm{NLS}_{\mathbf{a_3}}$ but equal phase at every lattice site (shown in the inset). (a2) Intensity after propagation of the diffracting state in the lattice. (b1)-(b2) Same as (a1)-(a2),but for the diffracting counterpart to $\mathrm{NLS}_{\mathbf{a_2}}$.

V. Numerically simulated propagation of a NLS and diffracting counterpart in the lattice

We perform continuous-model numerical simulations to corroborate our experimental observations. Therefore, we solve the z-propagation of a slowly varying envelope light field through a photonic lattice in the paraxial approximation via a standard split-step Fourier transform method.[2] The parameters of the simulations are adapted to match our experiments as close as possible. The waveguides are considered to have a refractive index contrast of $\Delta n = 1.1 \times 10^{-3}$ over the background with $n_0 = 1.5$. The slight ellipticity of the waveguides is compensated by a vertical stretch with a factor of 1.05 in analogy to the experiment.

The results of the simulations are presented in FIG. S4. If we excite the lattice with a light field corresponding to $\mathrm{NLS}_{\mathbf{a_2}}$, as can be seen in FIG. S4 (a), the intensity will stay completely localized to the initially excited waveguides even for much longer propagation distances of 8 cm. If we instead excite the same waveguides but without the π-phase difference needed to match the NLS, we will excite many modes with different propagation constants. As shown in FIG. S4 (b), this leads to diffraction over the whole lattice. By noticing how closely the diffracted pattern for a propagation distance of $z = 2\,\mathrm{cm}$ matches the experimentally

obtained one, we are able to confirm that our simulation parameters are well-calibrated and deliver an adequate model for the real lattice.

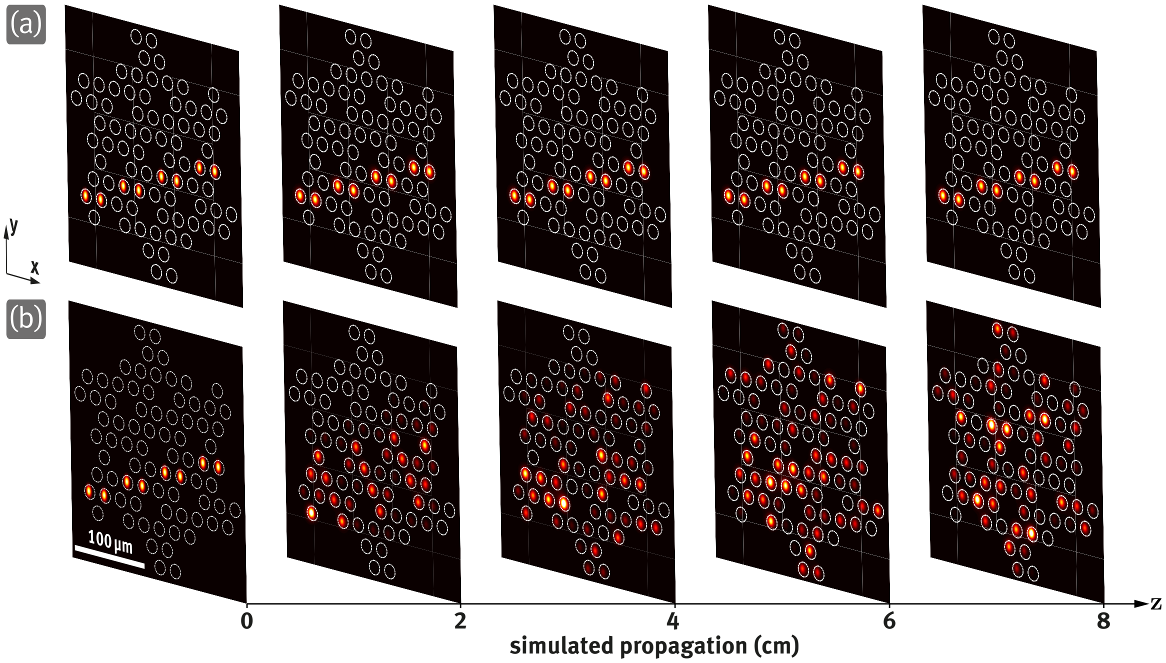

FIG. S4: Simulated propagation of $NLS_{\mathbf{a_1}}$ and its diffracting counterpart in the photonic borophene lattice. (a) Intensity of $NLS_{\mathbf{a_1}}$ for propagation distances of $z = 0, 2, 4, 6, 8\,\mathrm{cm}$. (b) same as (a), but for the diffracting counterpart having equal phase at every lattice site.

References

(1) Rhim, J.-W.; Yang, B.-J. Classification of flat bands according to the band-crossing singularity of Bloch wave functions. *Physical Review B* **2019**, *99*, 045107.

(2) Sharma, A.; Agrawal, A. New method for nonparaxial beam propagation. *JOSA A* **2004**, *21*, 1082–1087.
